# Supplementary material for: 7-O-tyrosyl Silybin Derivatives as a Novel Set of Anti-Prostate Cancer Compounds
Source: Antioxidants (Basel). 2024 Mar 29;13(4):418. doi: 10.3390/antiox13040418 (PMC11047488; doi:10.3390/antiox13040418)
Supplement: Supplementary file 1 [file antioxidants-13-00418-s001.zip › antioxidants-2913962-supplementary.pdf]

Supporting Information for

7-O-tyrosyl silybin derivatives as a novel set of anti-prostate cancer compounds.

Valeria Romanucci[a†], Rita Pagano[a†], Kushal Kandhari [b], Armando Zarrelli[a], Maria Petrone[a], Chapla Agarwal[b], Rajesh Agarwal[b] and Giovanni Di Fabio[a]*

[a] Department of Chemical Sciences, University of Naples "Federico II", Complesso Monte Sant'Angelo, Via Cintia 4, I-80126 Napoli (NA), Italy.

[b] University of Colorado, Skaggs School of Pharmacy and Pharmaceutical Sciences, Aurora, CO 80045, USA.

† These authors contributed equally to this work.

* corresponding author: Prof. Giovanni Di Fabio *e*mail: difabio@unina.it

Table of contents

	pag.
The ^{1}H and ^{13}C NMR data for compounds **10ab**	3S
The ^{1}H and ^{13}C NMR data for compound **10a**	5S
The ^{1}H and ^{13}C NMR data for compound **10b**	7S
The ^{1}H and ^{13}C NMR data for compounds **11ab**	9S
The ^{1}H and ^{13}C NMR data for compound **11a**	11S
The ^{1}H and ^{13}C NMR data for compound **11b**	13S
The ^{1}H and ^{13}C NMR data for compounds **12ab**	15S
The ^{1}H and ^{13}C NMR data for compound **12a**	17S
The ^{1}H and ^{13}C NMR data for compound **12b**	19S
The ^{1}H and ^{13}C NMR data for final compound **13a**	21S
The ^{1}H and ^{13}C NMR data for final compound **14a**	23S
The ^{1}H and ^{13}C NMR data for final compound **15a**	25S

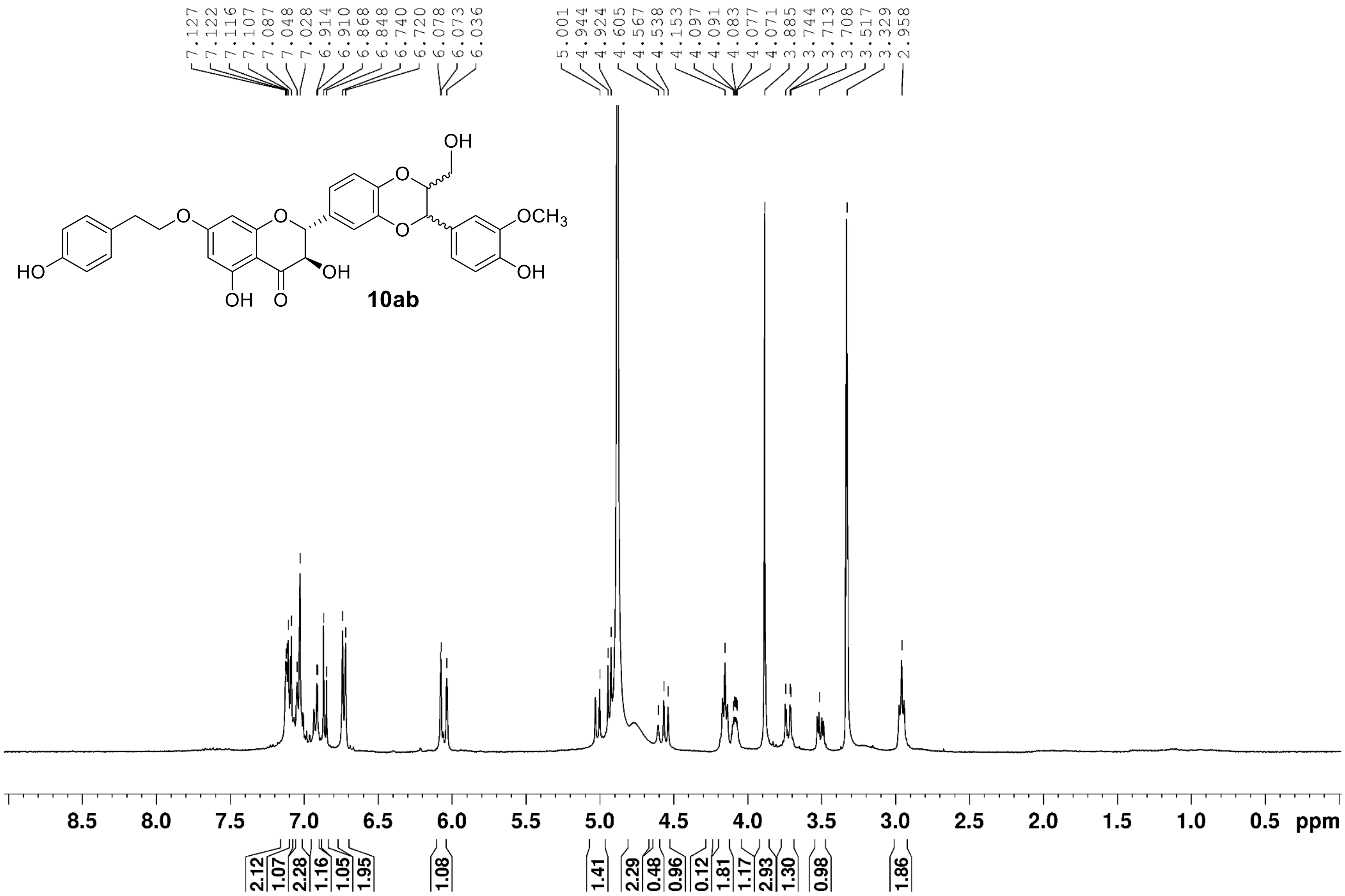
7.127 7.122 7.116 7.107 7.087 7.048 7.028 6.914 6.910 6.868 6.848 6.740 6.720 6.078 6.073 6.036
5.001 4.944 4.924 4.605 4.567 4.538 4.153 4.097 4.091 4.083 4.077 4.071 3.885 3.744 3.713 3.708 3.517 3.329 2.958
OH
O
O
OCH3
HO
OH
OH
O
OH
10ab
8.5 8.0 7.5 7.0 6.5 6.0 5.5 5.0 4.5 4.0 3.5 3.0 2.5 2.0 1.5 1.0 0.5 ppm
2.12 1.07 2.28 1.16 1.05 1.95 1.08 1.41 2.29 0.48 0.96 0.12 1.81 1.17 2.93 1.30 0.98 1.86

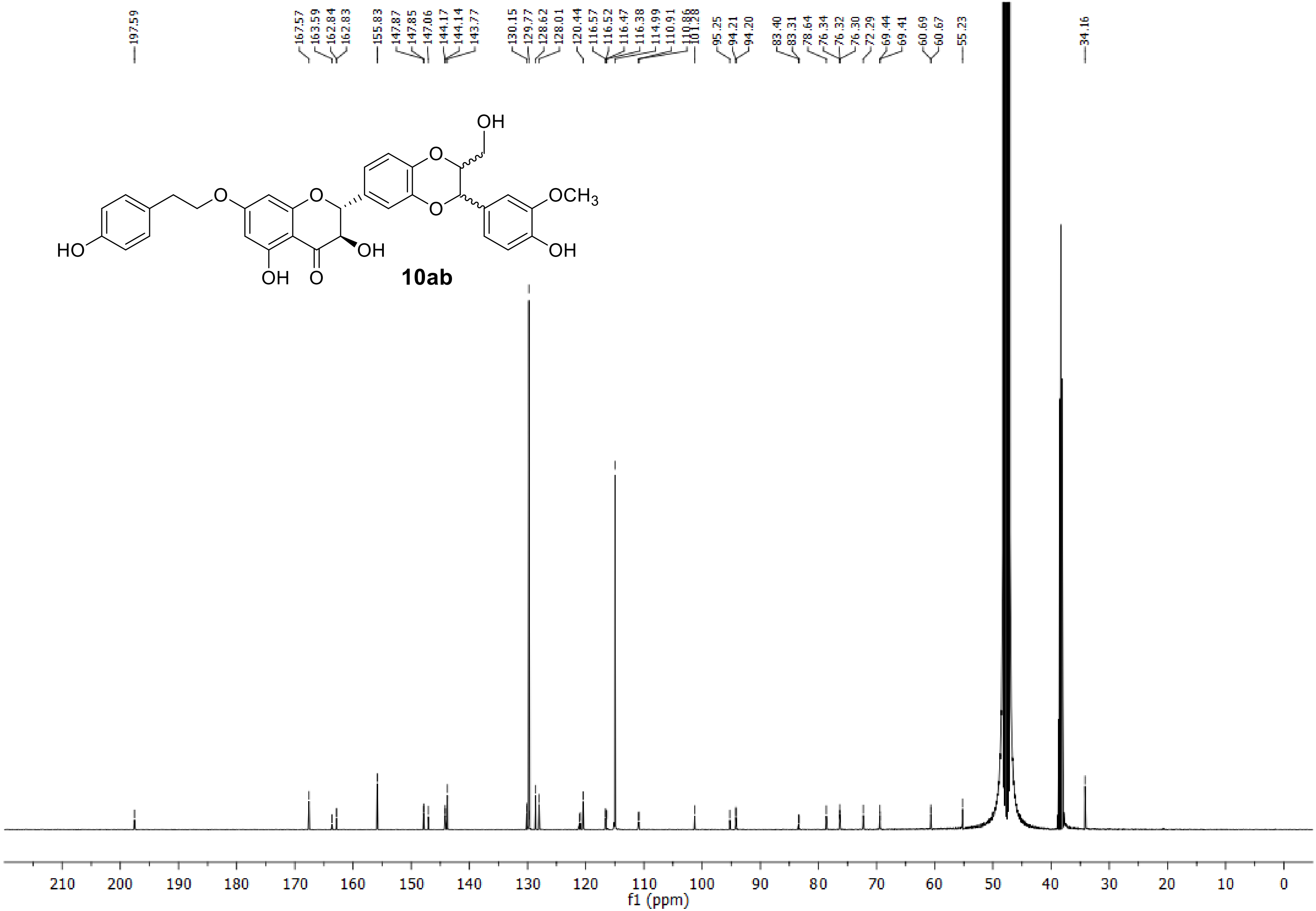
197.59
167.57
163.59
162.84
162.83
155.83
147.87
147.85
147.06
144.17
144.14
143.77
130.15
129.77
128.62
128.01
120.44
116.57
116.52
116.47
116.38
114.99
110.91
110.86
101.28
95.25
94.21
94.20
83.40
83.31
78.64
76.34
76.32
76.30
72.29
69.44
69.41
60.69
60.67
55.23
34.16
OH
O
O
O
OCH3
HO
OH
OH
OH
O
10ab
210
200
190
180
170
160
150
140
130
120
110
100
90
80
70
60
50
40
30
20
10
0
f1 (ppm)

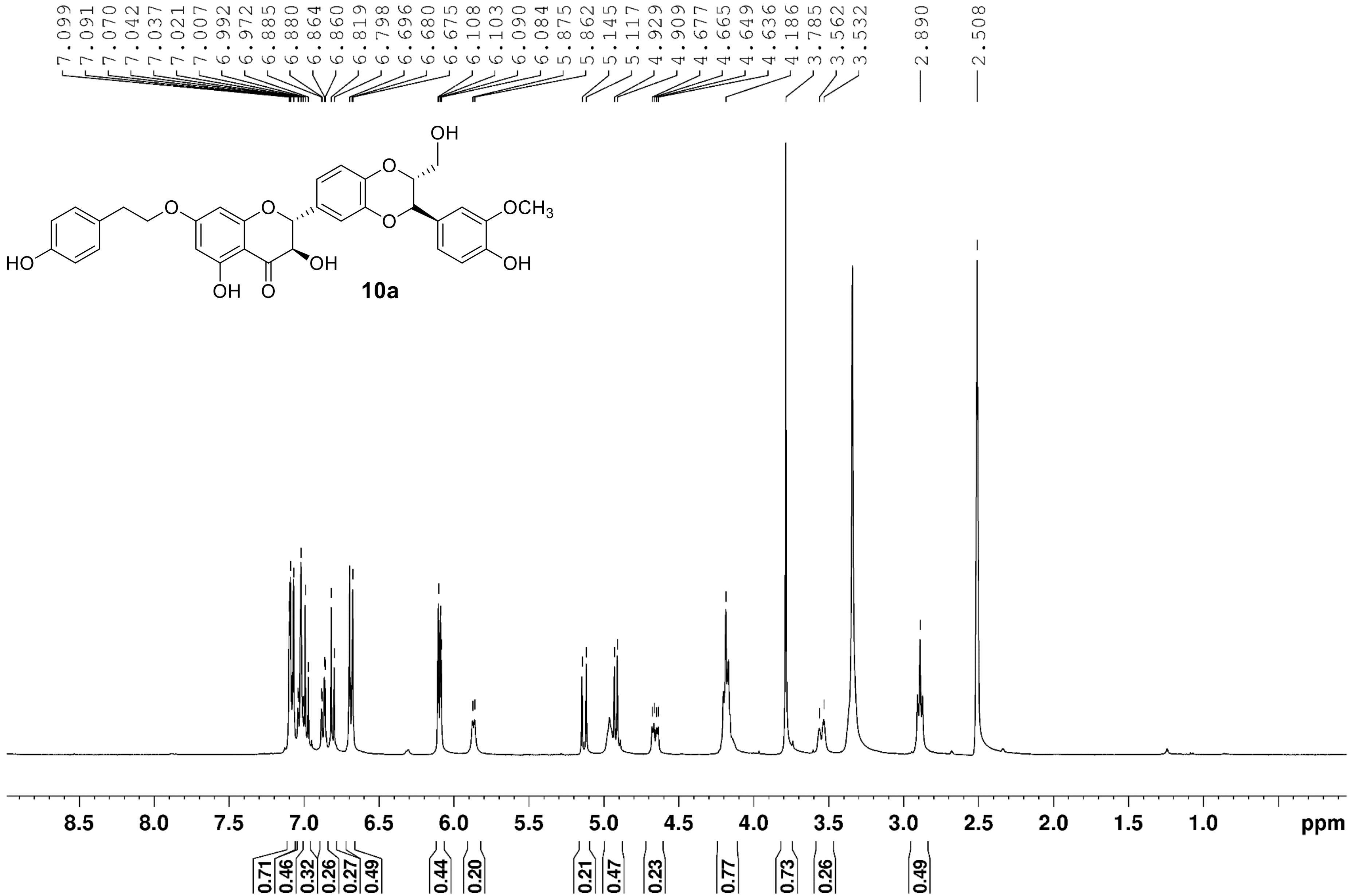
7.099
7.091
7.070
7.042
7.037
7.021
7.007
6.992
6.972
6.885
6.880
6.864
6.860
6.819
6.798
6.696
6.680
6.675
6.108
6.103
6.090
6.084
5.875
5.862
5.145
5.117
4.929
4.909
4.677
4.665
4.649
4.636
4.186
3.785
3.562
3.532
2.890
2.508
OH
OCH3
HO
OH
OH
OH
O
10a
8.5
8.0
7.5
7.0
6.5
6.0
5.5
5.0
4.5
4.0
3.5
3.0
2.5
2.0
1.5
1.0
ppm
0.71
0.46
0.32
0.26
0.27
0.49
0.44
0.20
0.21
0.47
0.23
0.77
0.73
0.26
0.49

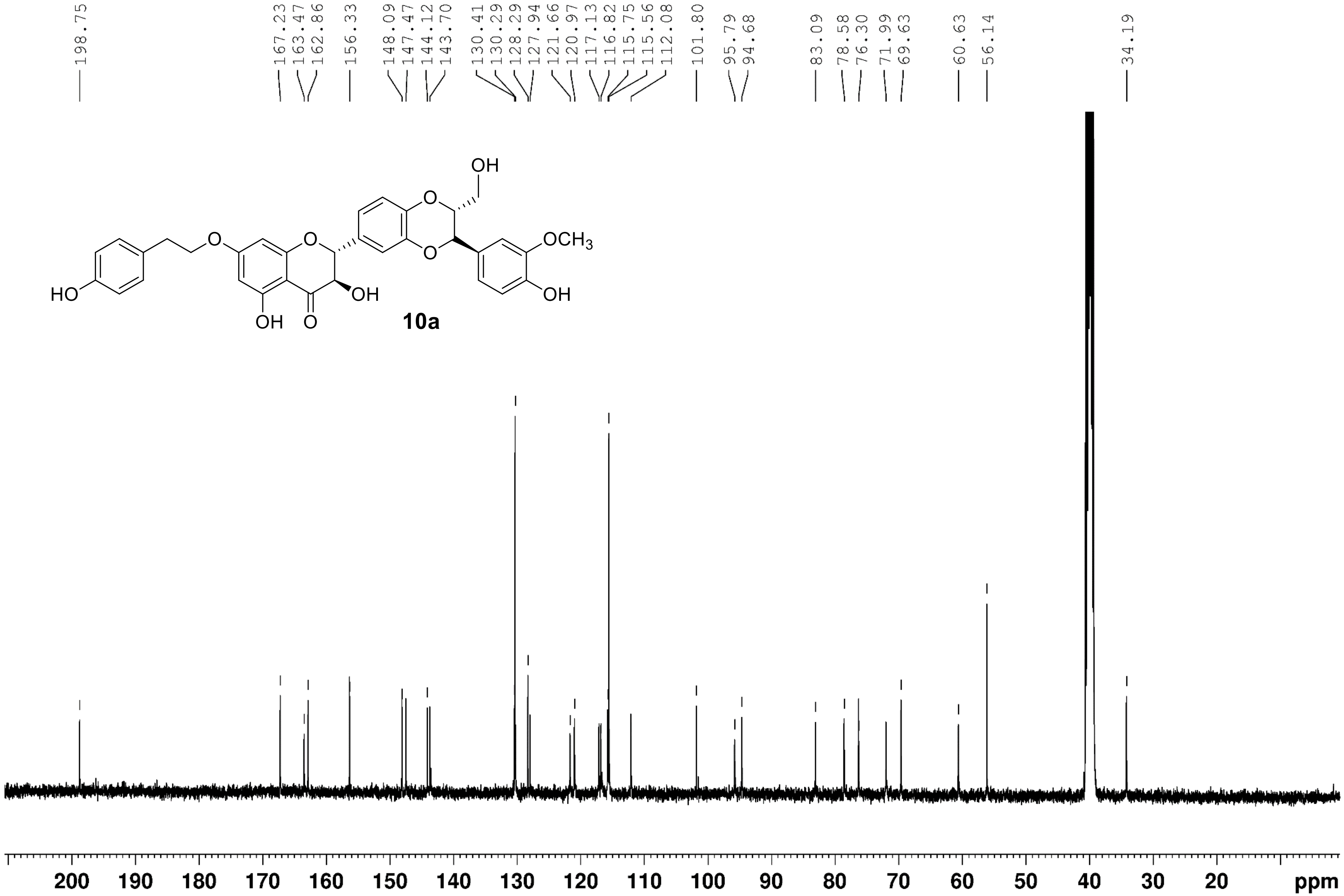

198.75
167.23
163.47
162.86
156.33
148.09
147.47
144.12
143.70
130.41
130.29
128.29
127.94
121.66
120.97
117.13
116.82
115.75
115.56
112.08
101.80
95.79
94.68
83.09
78.58
76.30
71.99
69.63
60.63
56.14
34.19
OH
OCH3
HO
OH
OH
OH
10a
200 190 180 170 160 150 140 130 120 110 100 90 80 70 60 50 40 30 20 ppm

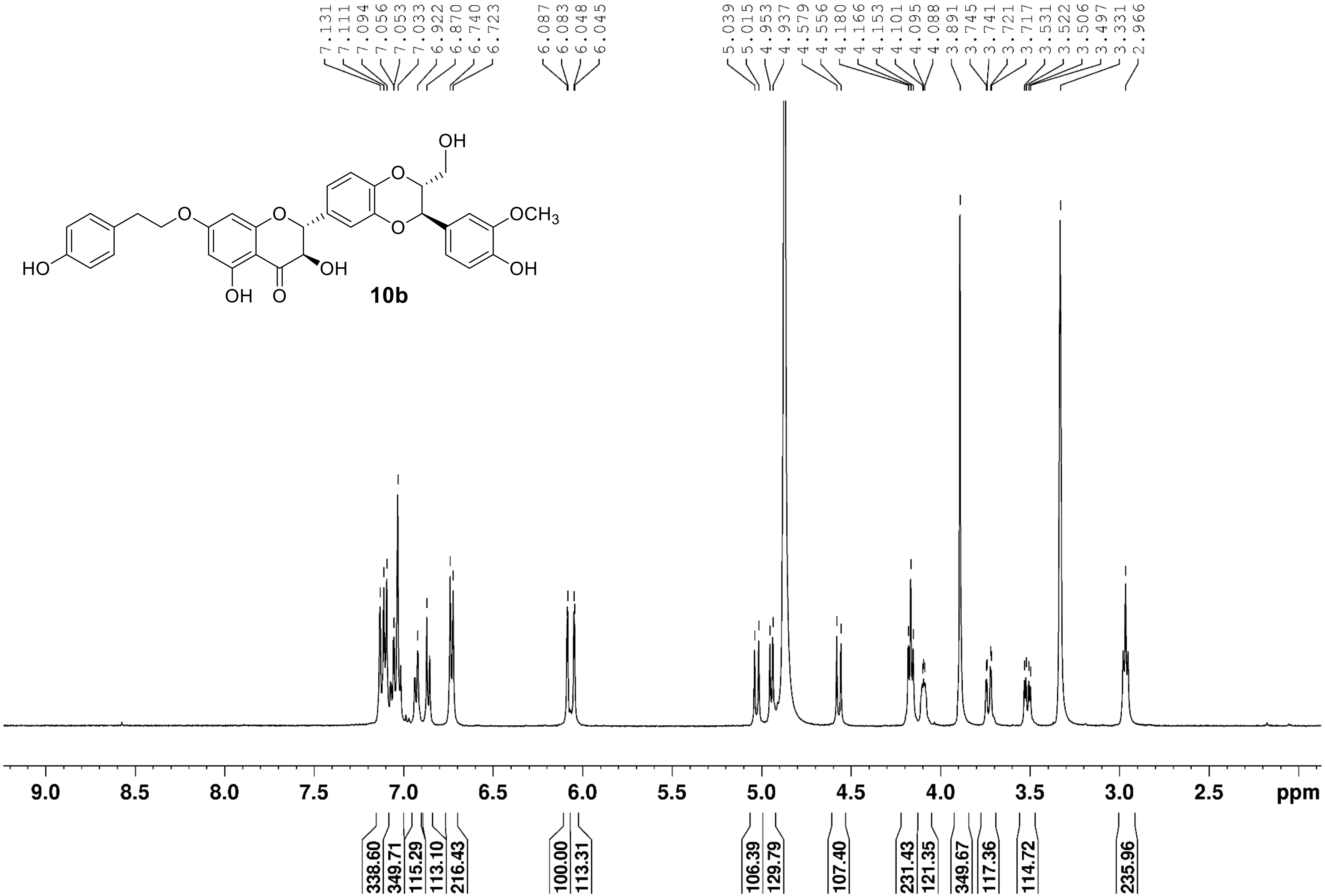
7.131
7.111
7.094
7.056
7.053
7.033
6.922
6.870
6.740
6.723
6.087
6.083
6.048
6.045
5.039
5.015
4.953
4.937
4.579
4.556
4.180
4.166
4.153
4.101
4.095
4.088
3.891
3.745
3.741
3.721
3.717
3.531
3.522
3.506
3.497
3.331
2.966
OH
OCH3
HO
OH
OH
O
OH
10b
9.0
8.5
8.0
7.5
7.0
6.5
6.0
5.5
5.0
4.5
4.0
3.5
3.0
2.5
ppm
338.60
349.71
115.29
113.10
216.43
100.00
113.31
106.39
129.79
107.40
231.43
121.35
349.67
117.36
114.72
235.96

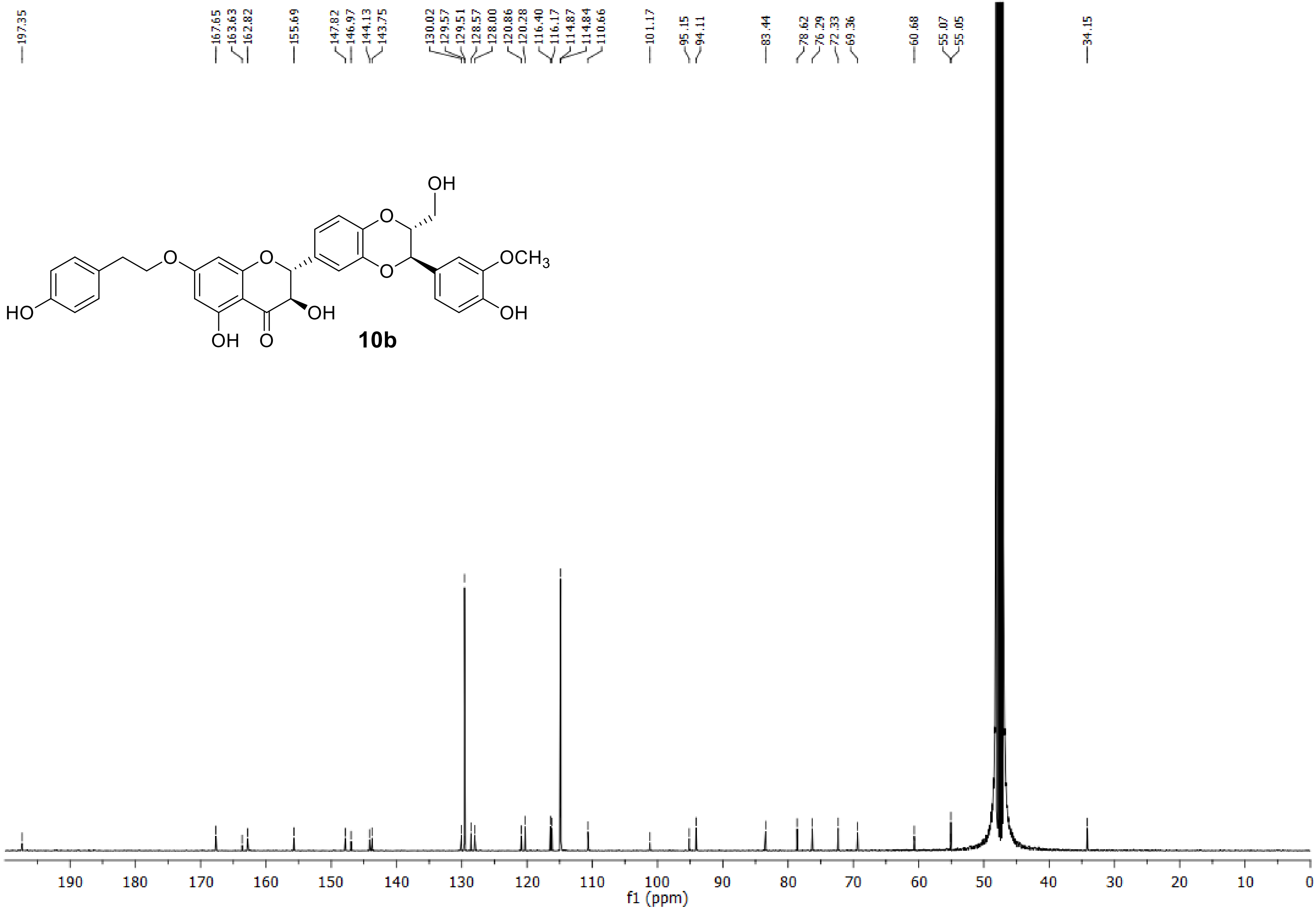
197.35
167.65
163.63
162.82
155.69
147.82
146.97
144.13
143.75
130.02
129.57
129.51
128.57
128.00
120.86
120.28
116.40
116.17
114.87
114.84
110.66
101.17
95.15
94.11
83.44
78.62
76.29
72.33
69.36
60.68
55.07
55.05
34.15
OH
O
O
OCH3
HO
OH
OH
OH O
10b
190 180 170 160 150 140 130 120 110 100 90 80 70 60 50 40 30 20 10 0
f1 (ppm)

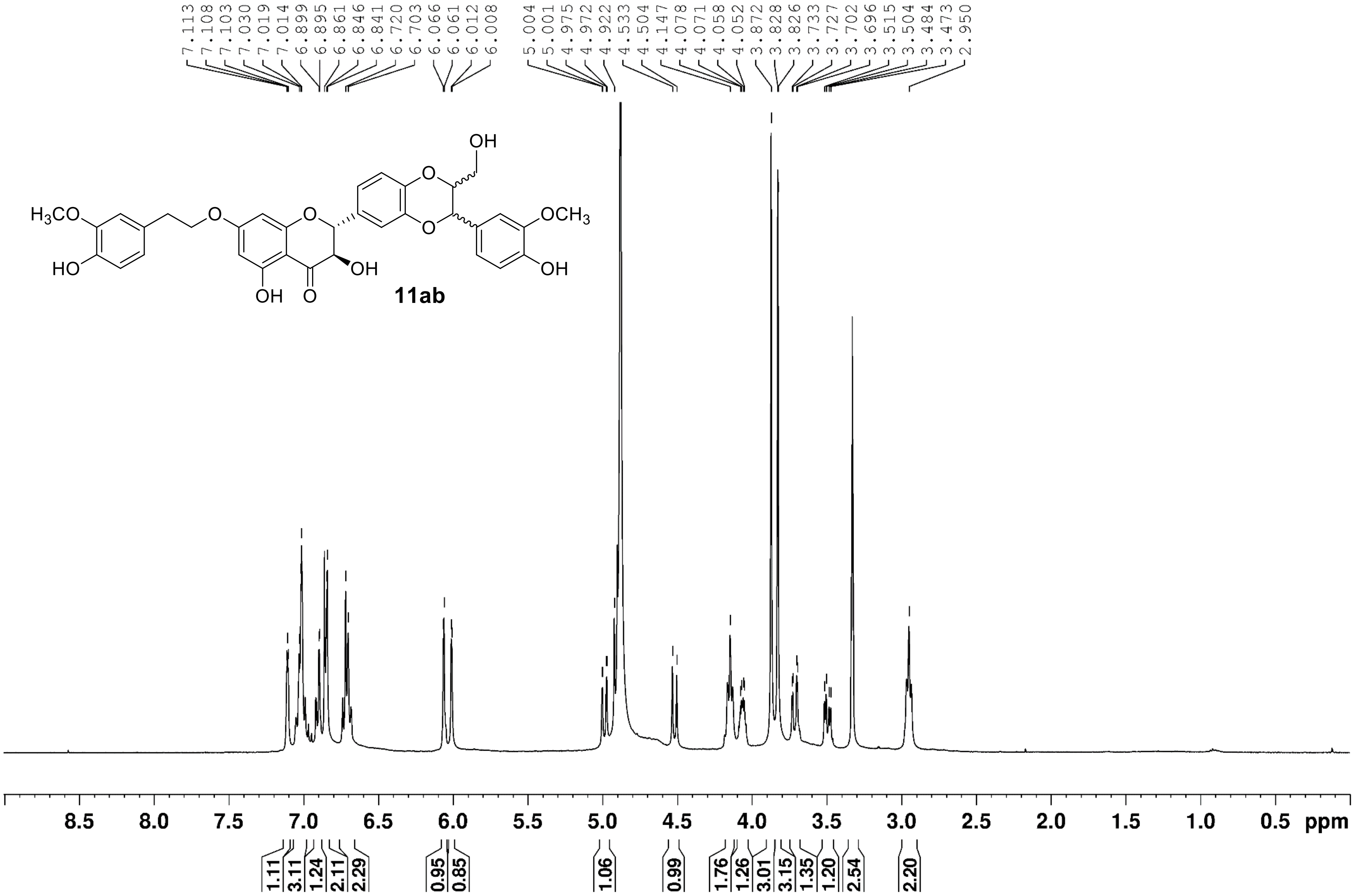

7.113
7.108
7.103
7.030
7.019
7.014
6.899
6.895
6.861
6.846
6.841
6.720
6.703
6.066
6.061
6.012
6.008
5.004
5.001
4.975
4.972
4.922
4.533
4.504
4.147
4.078
4.071
4.058
4.052
3.872
3.828
3.826
3.733
3.727
3.702
3.696
3.515
3.504
3.484
3.473
2.950
H3CO
HO
OH
OCH3
11ab
8.5
8.0
7.5
7.0
6.5
6.0
5.5
5.0
4.5
4.0
3.5
3.0
2.5
2.0
1.5
1.0
0.5
ppm
1.11
3.11
1.24
2.11
2.29
0.95
0.85
1.06
0.99
1.76
1.26
3.01
3.15
1.35
1.20
2.54
2.20

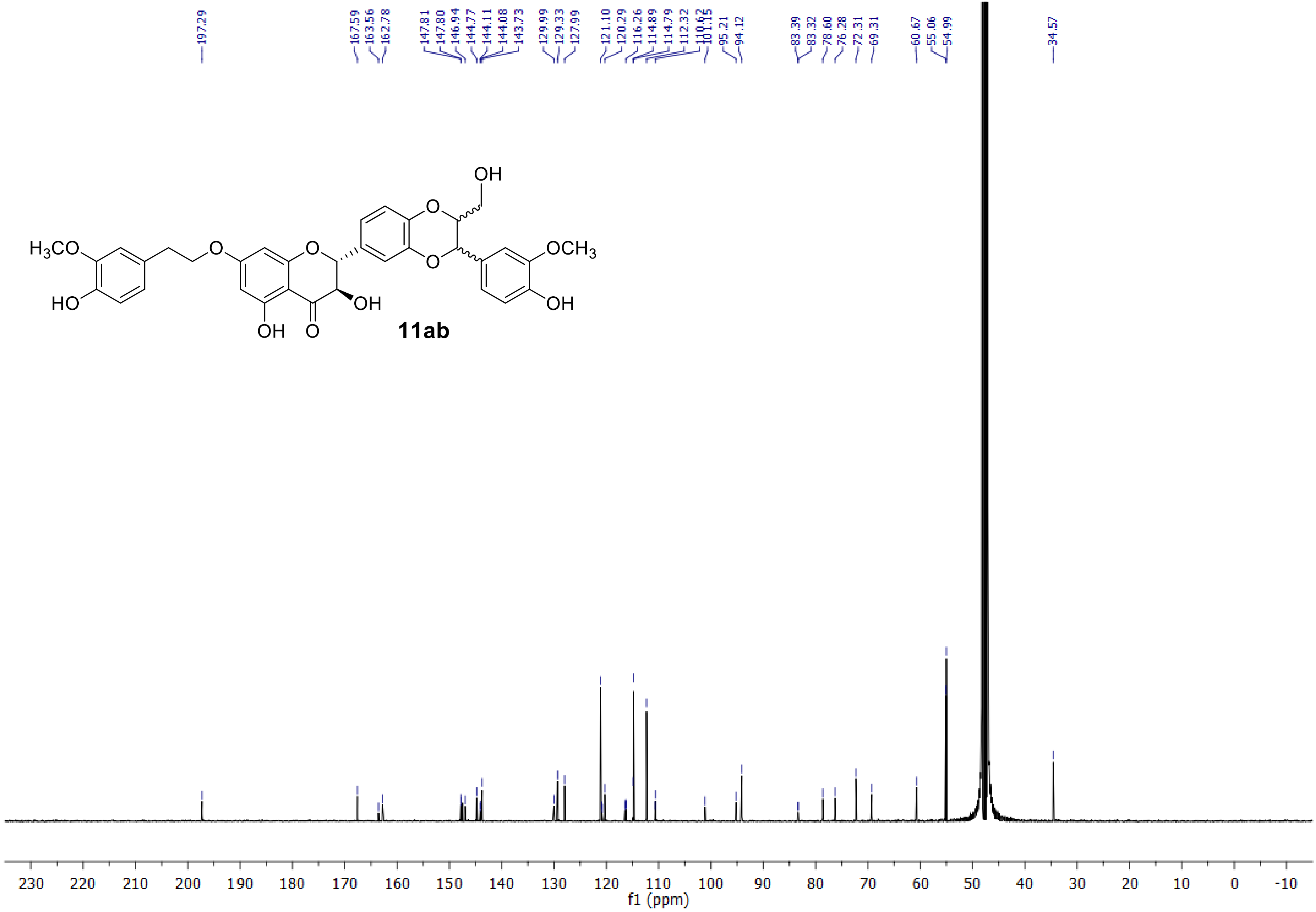
197.29
167.59
163.56
162.78
147.81
147.80
146.94
144.77
144.11
144.08
143.73
129.99
129.33
127.99
121.10
120.29
116.26
114.89
114.79
112.32
110.62
101.15
95.21
94.12
83.39
83.32
78.60
76.28
72.31
69.31
60.67
55.06
54.99
34.57
OH
O
O
H_3CO
O
O
OCH_3
HO
OH
OH
OH
O
11ab
230 220 210 200 190 180 170 160 150 140 130 120 110 100 90 80 70 60 50 40 30 20 10 0 -10
f1 (ppm)

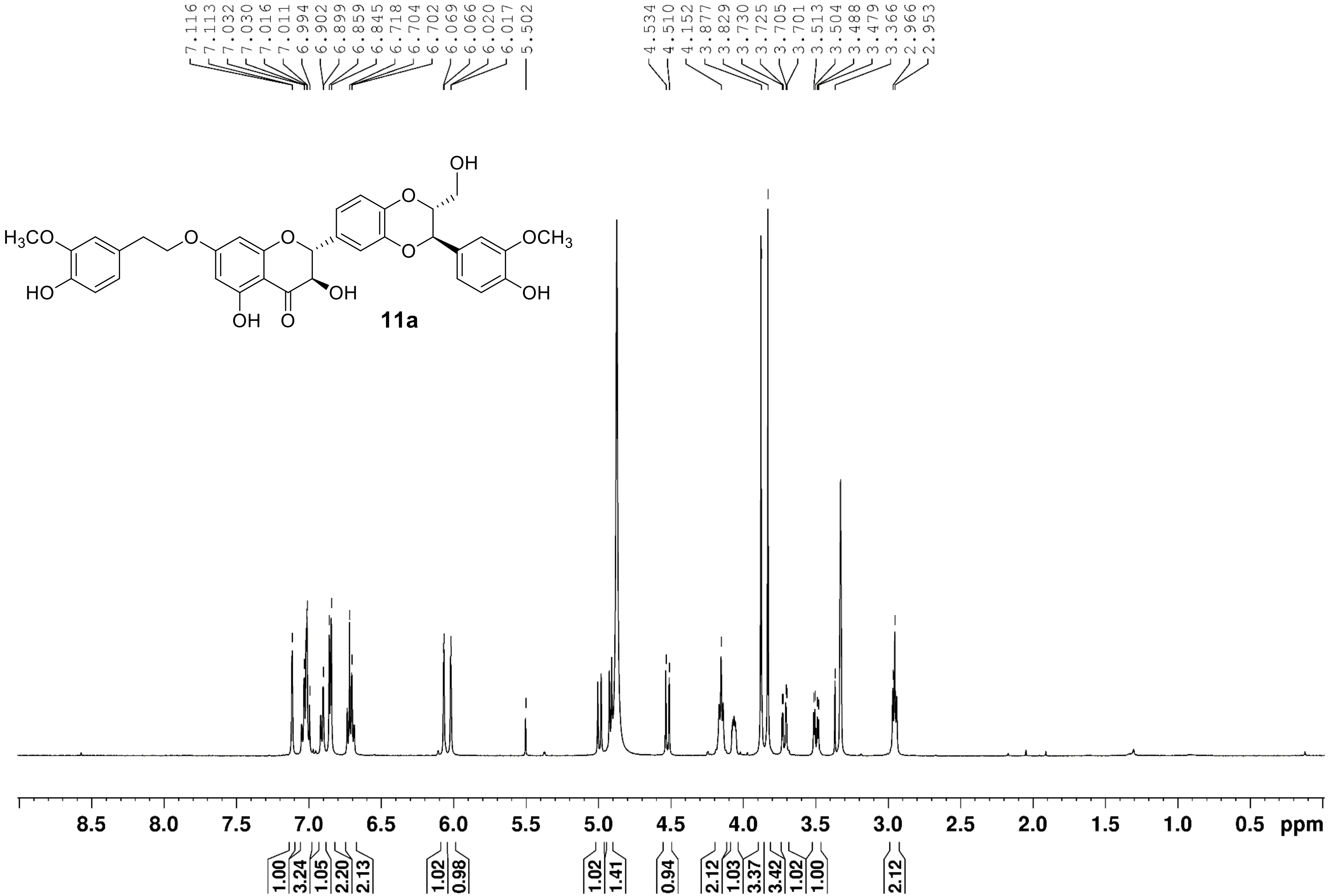
7.116
7.113
7.032
7.030
7.016
7.011
6.994
6.902
6.899
6.859
6.845
6.718
6.704
6.702
6.069
6.066
6.020
6.017
5.502
4.534
4.510
4.152
3.877
3.829
3.730
3.725
3.705
3.701
3.513
3.504
3.488
3.479
3.366
2.966
2.953
OH
O
O
H_3CO
O
O
OCH_3
HO
OH
OH
OH
O
11a
8.5
8.0
7.5
7.0
6.5
6.0
5.5
5.0
4.5
4.0
3.5
3.0
2.5
2.0
1.5
1.0
0.5
ppm
1.00
3.24
1.05
2.20
2.13
1.02
0.98
1.02
1.41
0.94
2.12
1.03
3.37
3.42
1.02
1.00
2.12

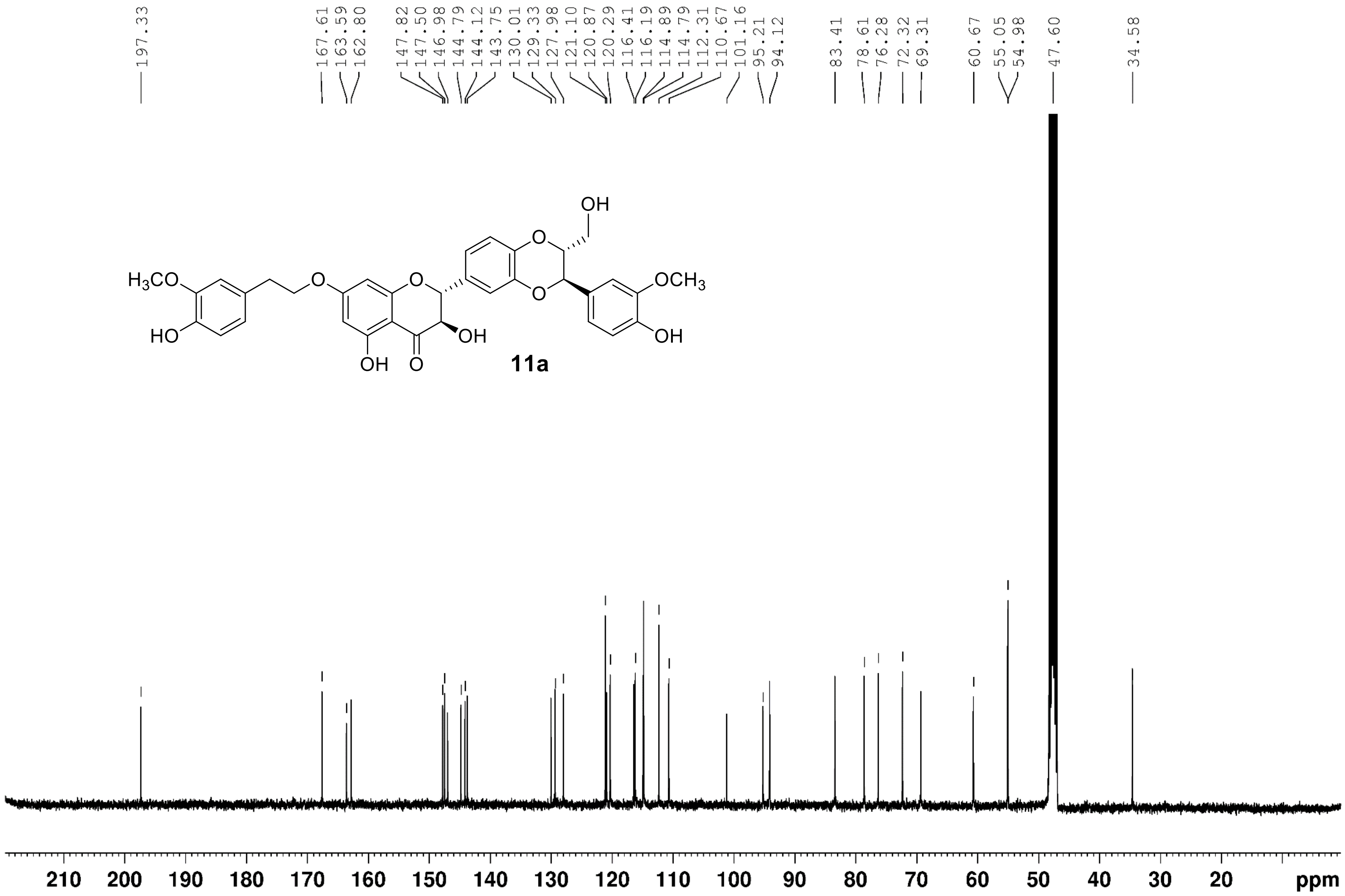
197.33
167.61
163.59
162.80
147.82
147.50
146.98
144.79
144.12
143.75
130.01
129.33
127.98
121.10
120.87
120.29
116.41
116.19
114.89
114.79
112.31
110.67
101.16
95.21
94.12
83.41
78.61
76.28
72.32
69.31
60.67
55.05
54.98
47.60
34.58
H3CO
HO
O
O
OH
OH
O
O
O
OH
OCH3
OH
11a
210
200
190
180
170
160
150
140
130
120
110
100
90
80
70
60
50
40
30
20
ppm

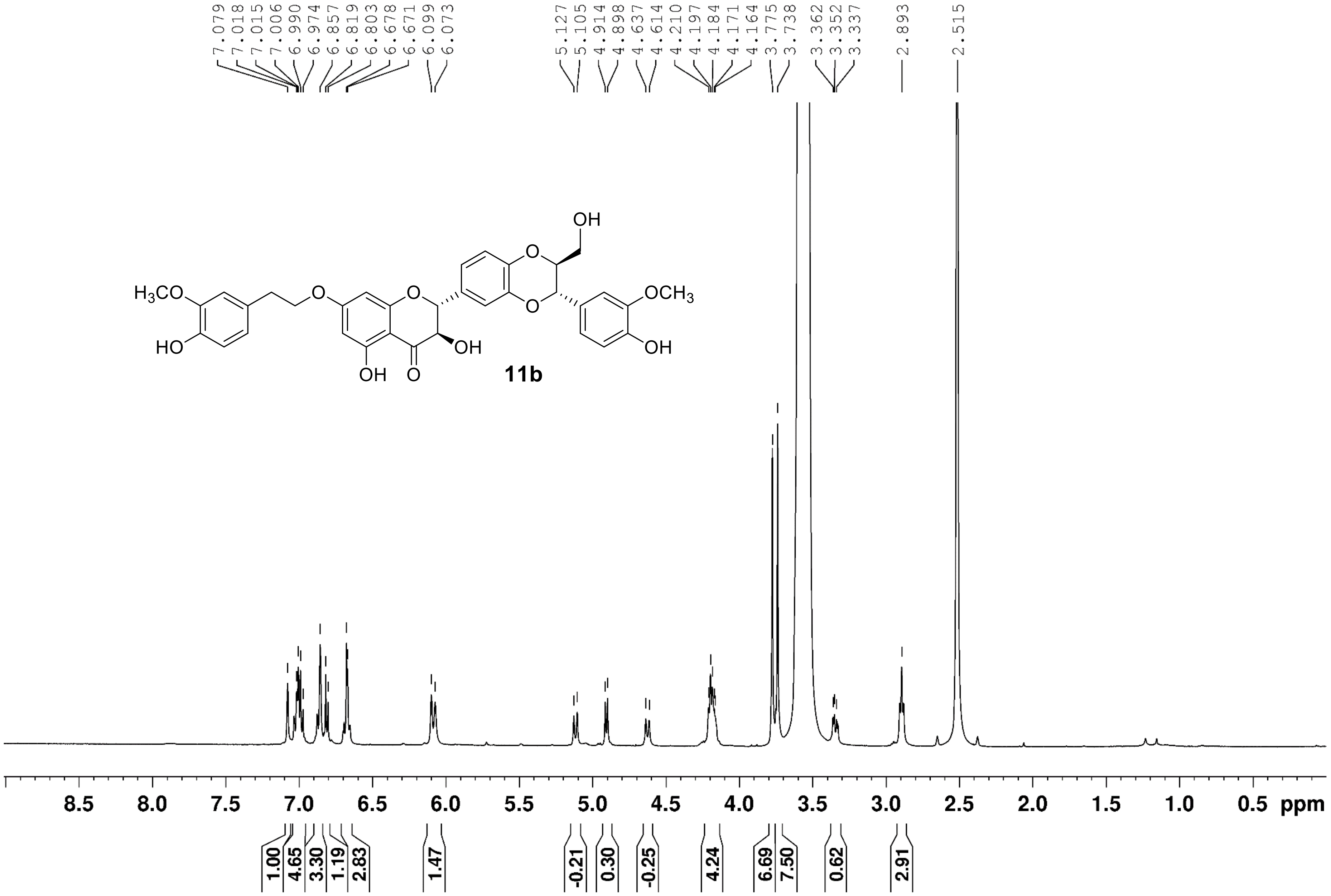
7.079
7.018
7.015
7.006
6.990
6.974
6.857
6.819
6.803
6.678
6.671
6.099
6.073
5.127
5.105
4.914
4.898
4.637
4.614
4.210
4.197
4.184
4.171
4.164
3.775
3.738
3.362
3.352
3.337
2.893
2.515
H3CO
HO
OH
O
OCH3
11b
8.5
8.0
7.5
7.0
6.5
6.0
5.5
5.0
4.5
4.0
3.5
3.0
2.5
2.0
1.5
1.0
0.5
ppm
1.00
4.65
3.30
1.19
2.83
1.47
-0.21
0.30
-0.25
4.24
6.69
7.50
0.62
2.91

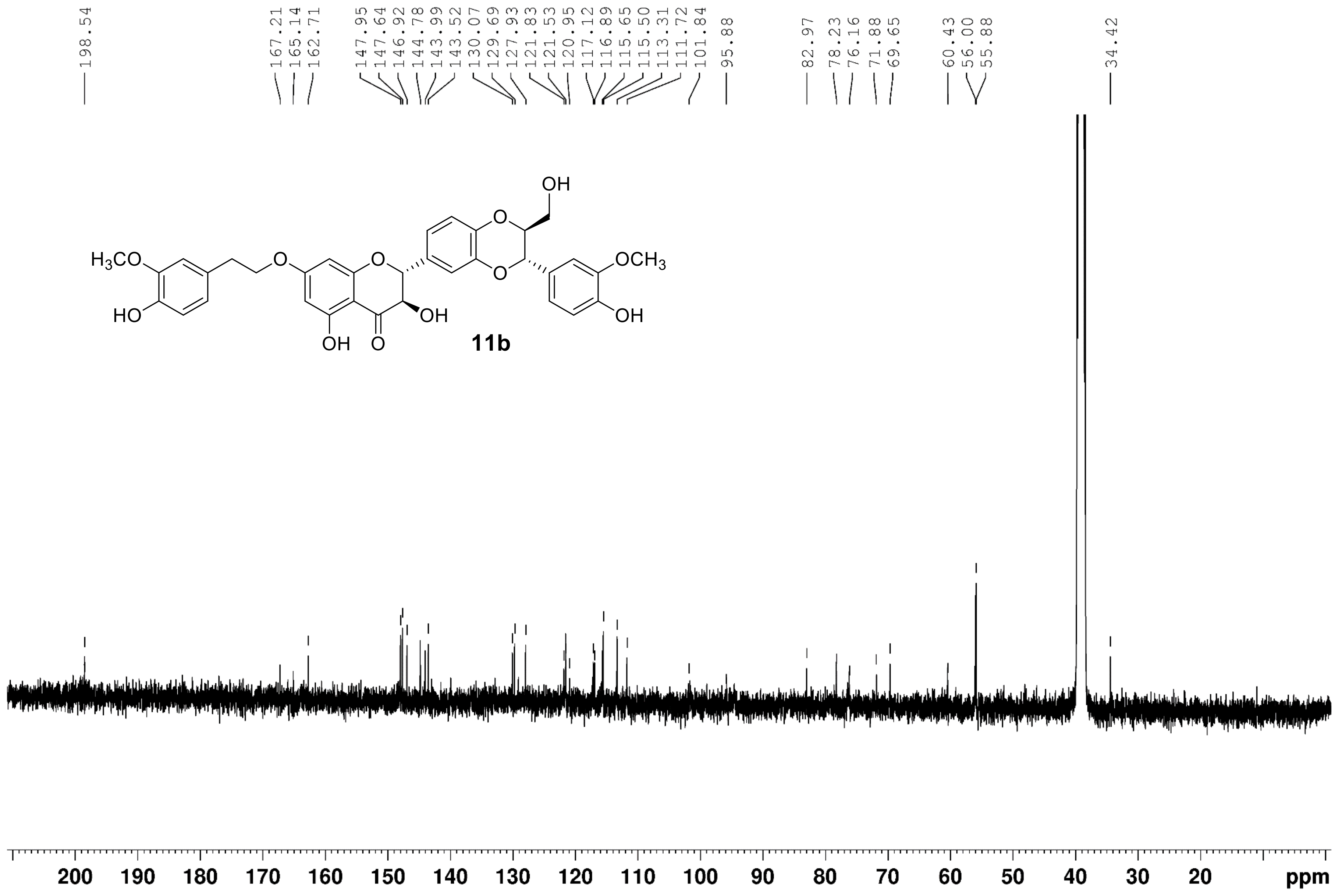
198.54
167.21
165.14
162.71
147.95
147.64
146.92
144.78
143.99
143.52
130.07
129.69
127.93
121.83
121.53
120.95
117.12
116.89
115.65
115.50
113.31
111.72
101.84
95.88
82.97
78.23
76.16
71.88
69.65
60.43
56.00
55.88
34.42
OH
O
H3CO
O
O
O
OCH3
HO
OH
OH
OH
O
11b
200 190 180 170 160 150 140 130 120 110 100 90 80 70 60 50 40 30 20 ppm

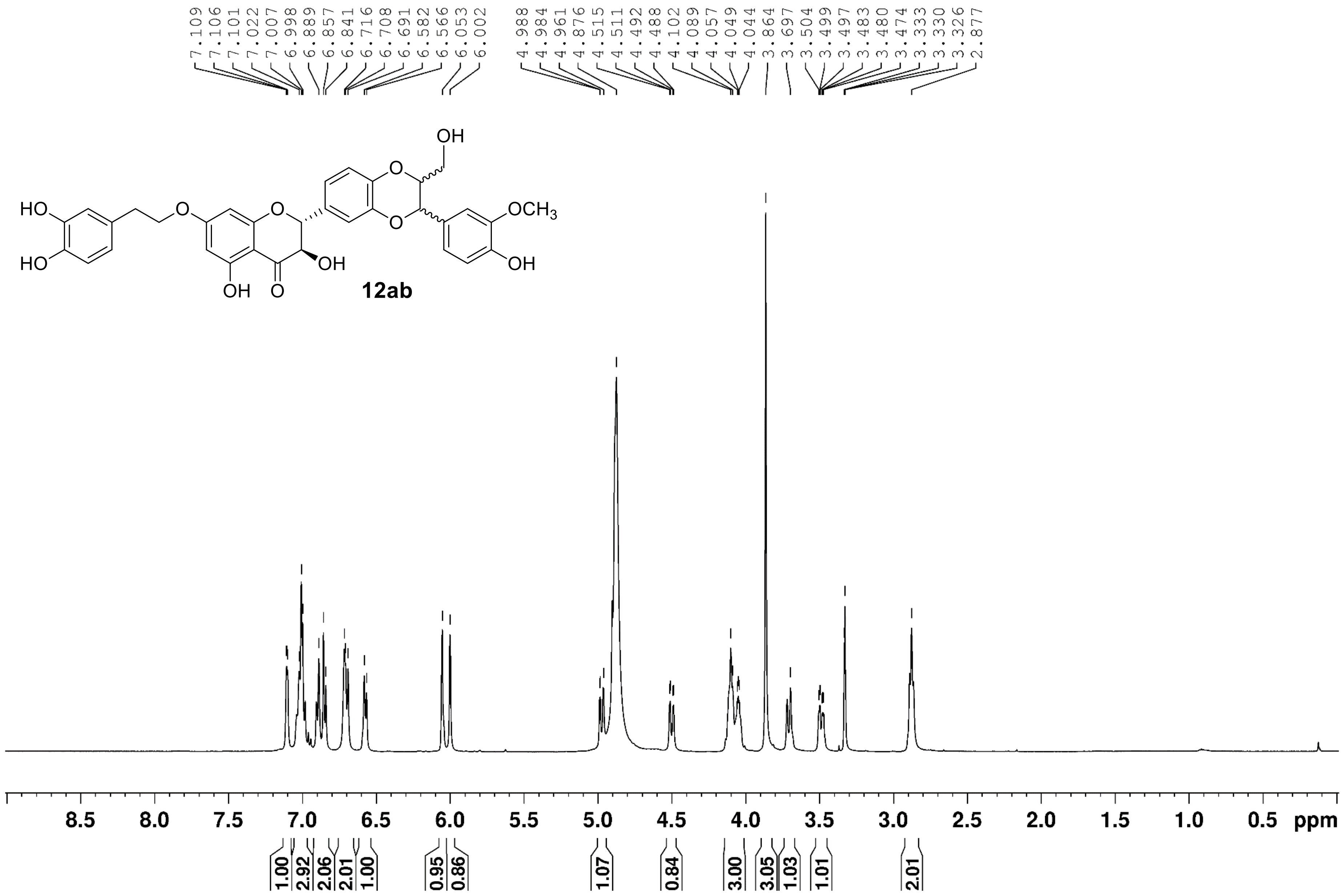
7.109
7.106
7.101
7.022
7.007
6.998
6.889
6.857
6.841
6.716
6.708
6.691
6.582
6.566
6.053
6.002
4.988
4.984
4.961
4.876
4.515
4.511
4.492
4.488
4.102
4.089
4.057
4.049
4.044
3.864
3.697
3.504
3.499
3.497
3.483
3.480
3.474
3.333
3.330
3.326
2.877
HO
HO
O
O
OH
O
OH
O
O
OH
OCH3
OH
12ab
8.5
8.0
7.5
7.0
6.5
6.0
5.5
5.0
4.5
4.0
3.5
3.0
2.5
2.0
1.5
1.0
0.5
ppm
1.00
2.92
2.06
2.01
1.00
0.95
0.86
1.07
0.84
3.00
3.05
1.03
1.01
2.01

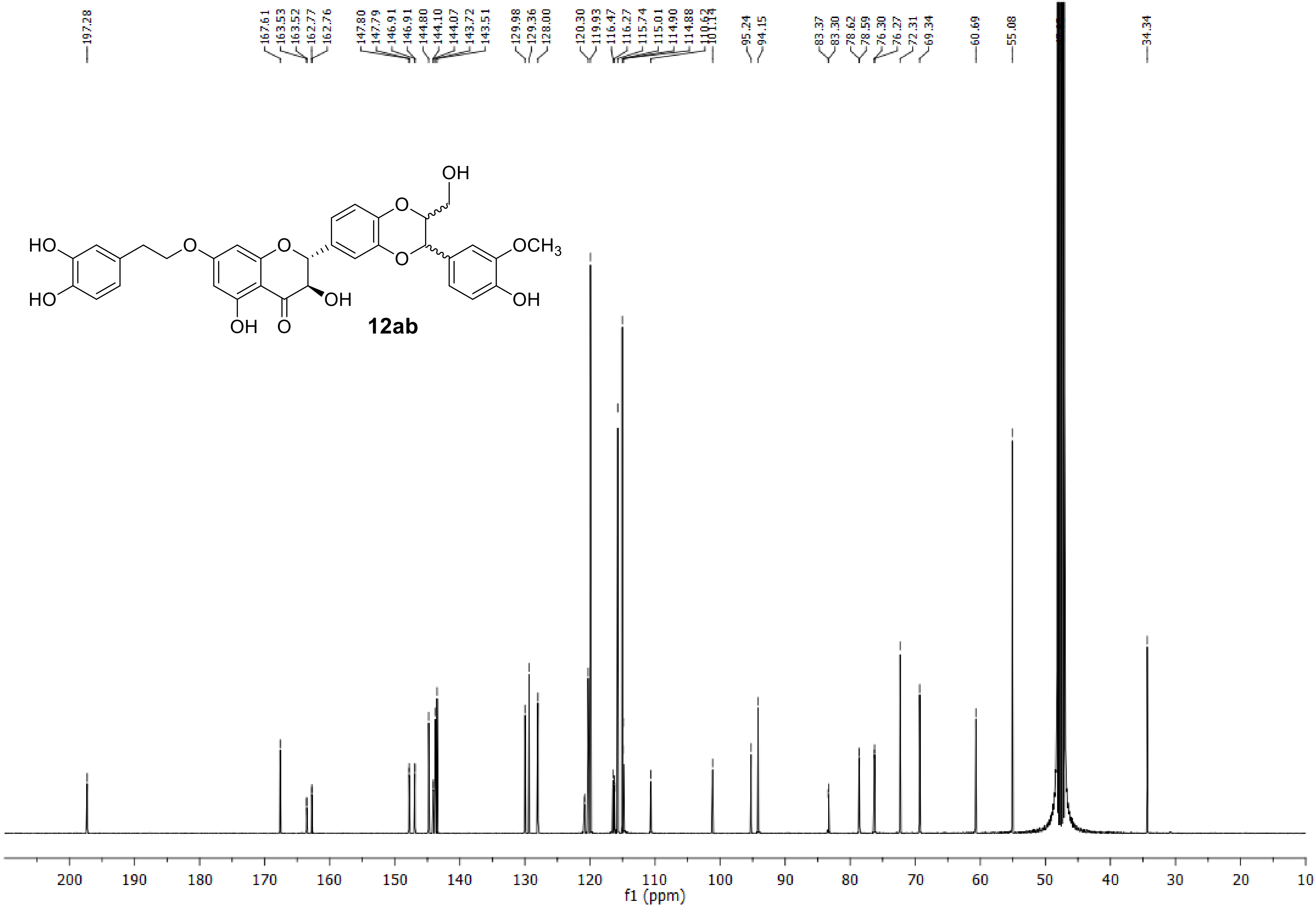

HO
HO
O
O
OH
O
OH
O
O
OH
OCH3
OH
12ab
f1 (ppm)

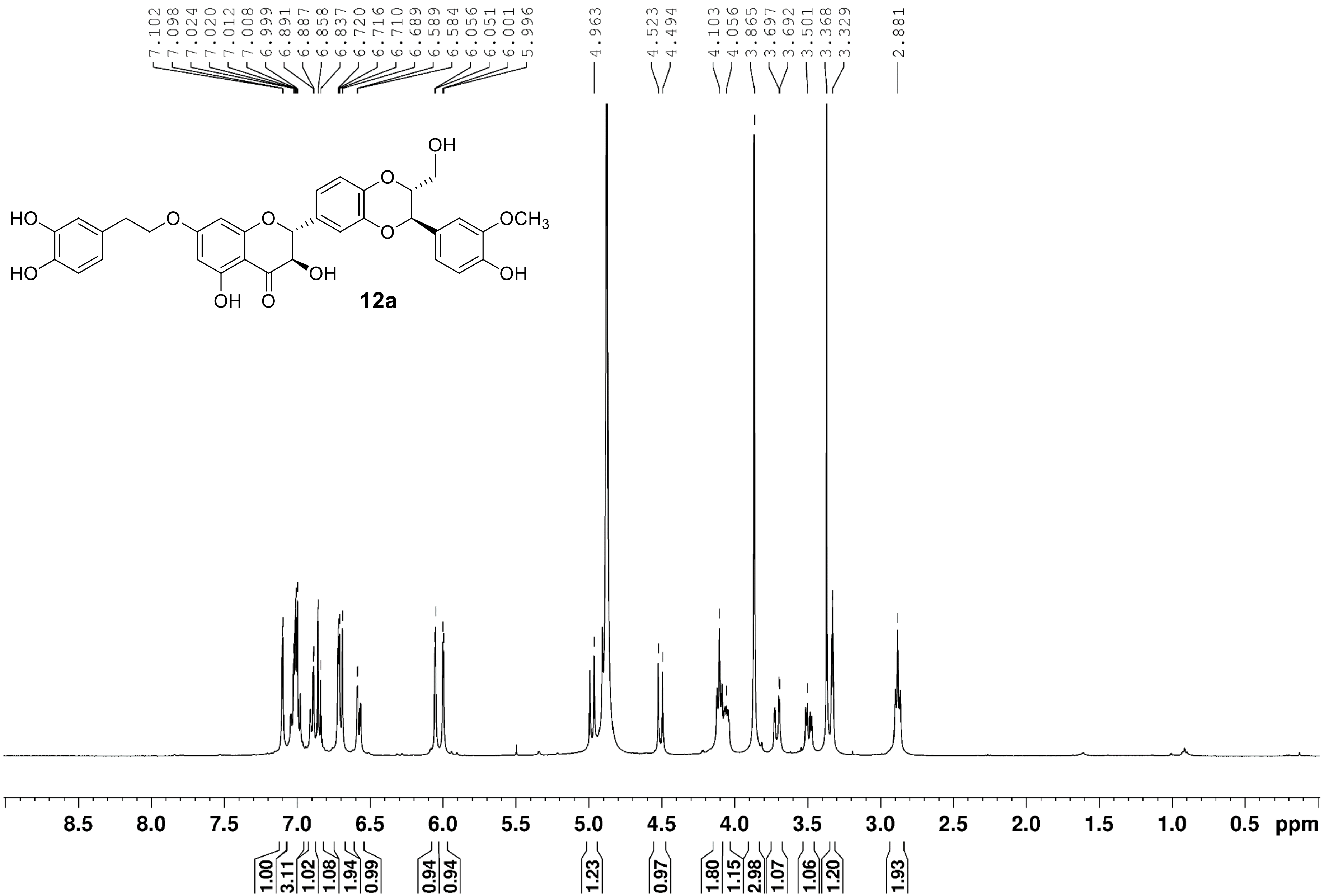

7.102
7.098
7.024
7.020
7.012
7.008
6.999
6.891
6.887
6.858
6.837
6.720
6.716
6.710
6.689
6.589
6.584
6.056
6.051
6.001
5.996
4.963
4.523
4.494
4.103
4.056
3.865
3.697
3.692
3.501
3.368
3.329
2.881
HO
HO
OH
OH
O
OH
O
O
O
OCH3
OH
12a
8.5
8.0
7.5
7.0
6.5
6.0
5.5
5.0
4.5
4.0
3.5
3.0
2.5
2.0
1.5
1.0
0.5
ppm
1.00
3.11
1.02
1.08
1.94
0.99
0.94
0.94
1.23
0.97
1.80
1.15
2.98
1.07
1.06
1.20
1.93

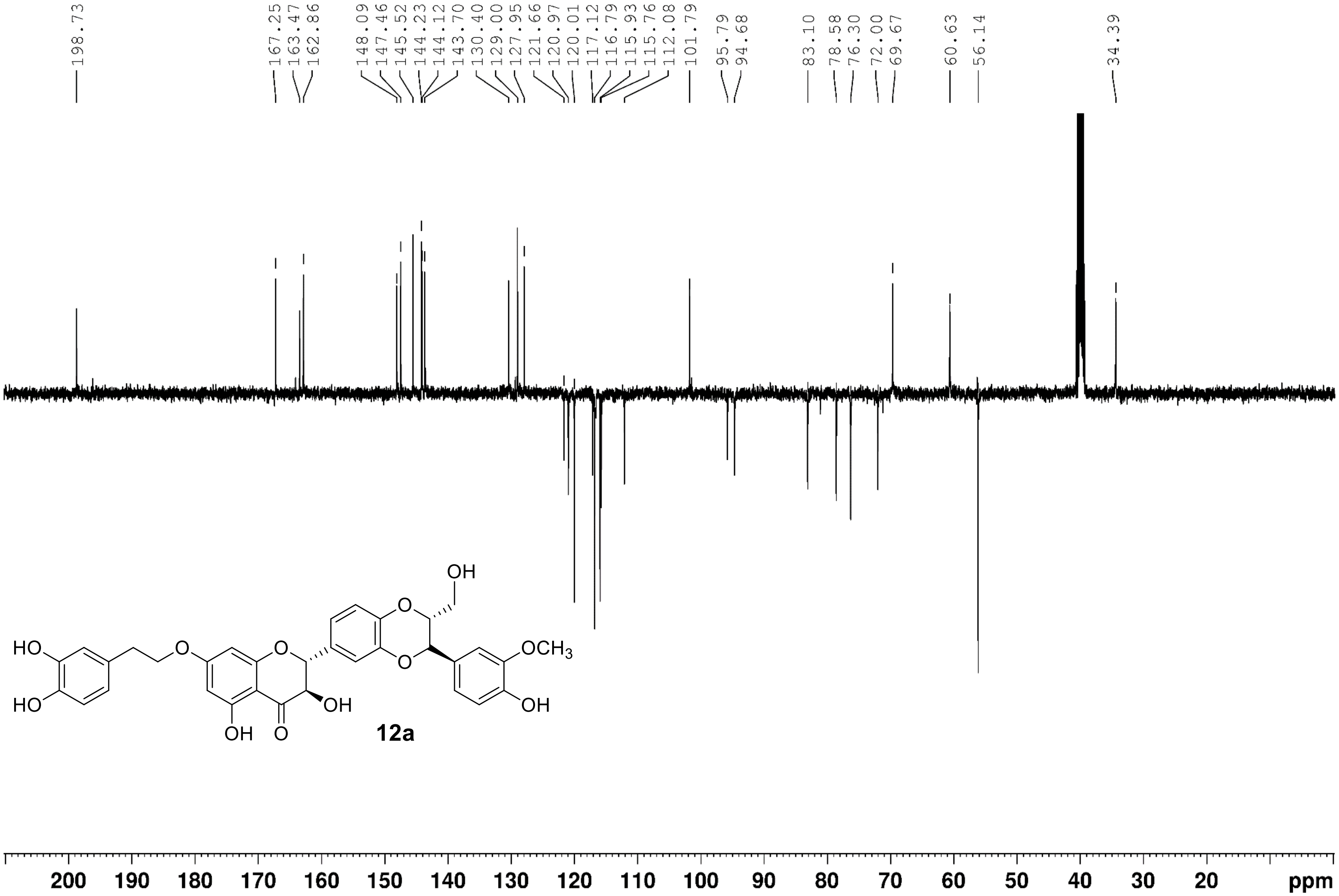
198.73
167.25
163.47
162.86
148.09
147.46
145.52
144.23
144.12
143.70
130.40
129.00
127.95
121.66
120.97
120.01
117.12
116.79
115.93
115.76
112.08
101.79
95.79
94.68
83.10
78.58
76.30
72.00
69.67
60.63
56.14
34.39
HO
HO
O
O
OH
O
O
OH
OCH3
OH
OH
O
OH
200 190 180 170 160 150 140 130 120 110 100 90 80 70 60 50 40 30 20 ppm

12a

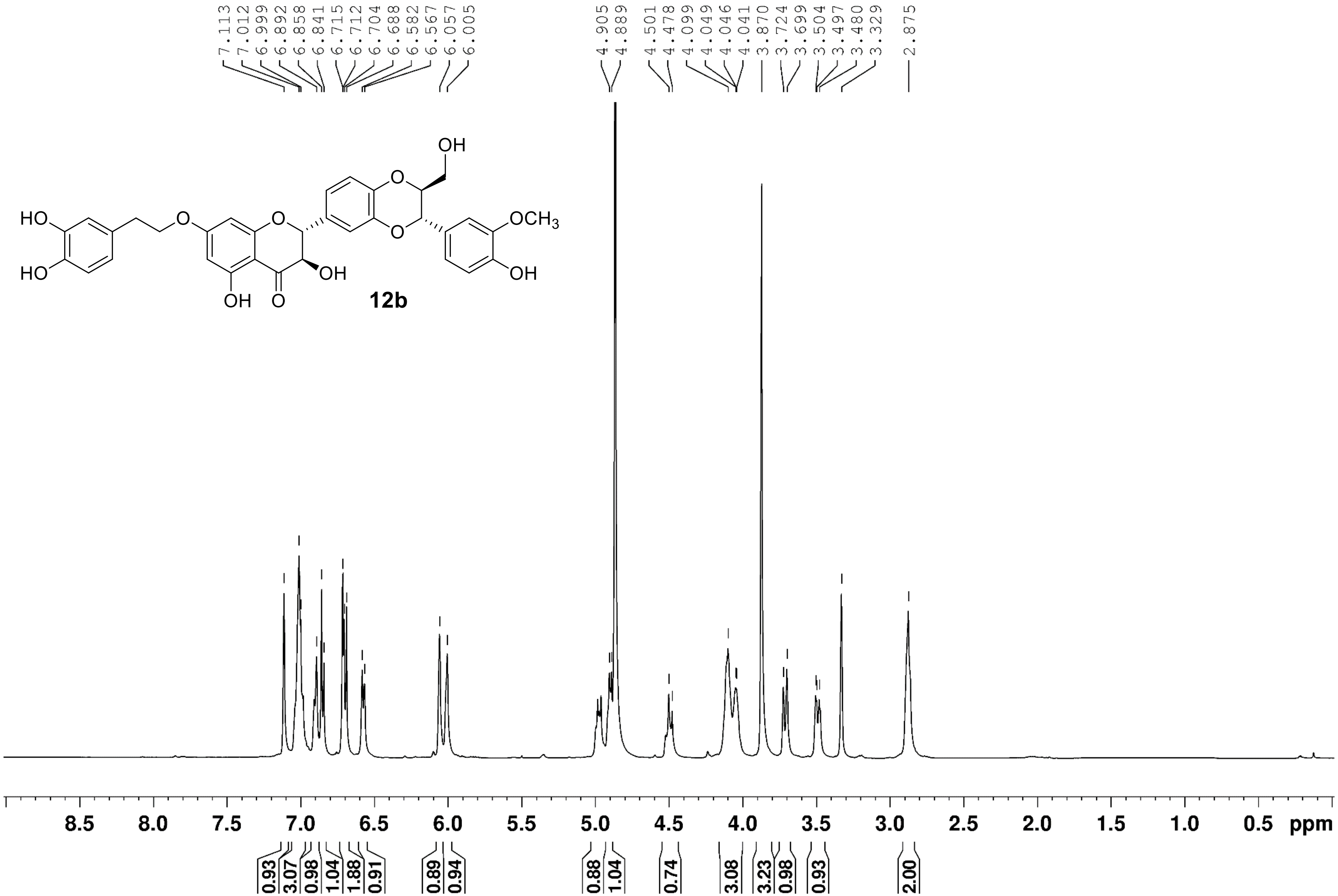
7.113
7.012
6.999
6.892
6.858
6.841
6.715
6.712
6.704
6.688
6.582
6.567
6.057
6.005
4.905
4.889
4.501
4.478
4.099
4.049
4.046
4.041
3.870
3.724
3.699
3.504
3.497
3.480
3.329
2.875
HO
HO
O
O
OH
OH
O
O
O
OH
OCH3
OH
12b
8.5
8.0
7.5
7.0
6.5
6.0
5.5
5.0
4.5
4.0
3.5
3.0
2.5
2.0
1.5
1.0
0.5
ppm
0.93
3.07
0.98
1.04
1.88
0.91
0.89
0.94
0.88
1.04
0.74
3.08
3.23
0.98
0.93
2.00

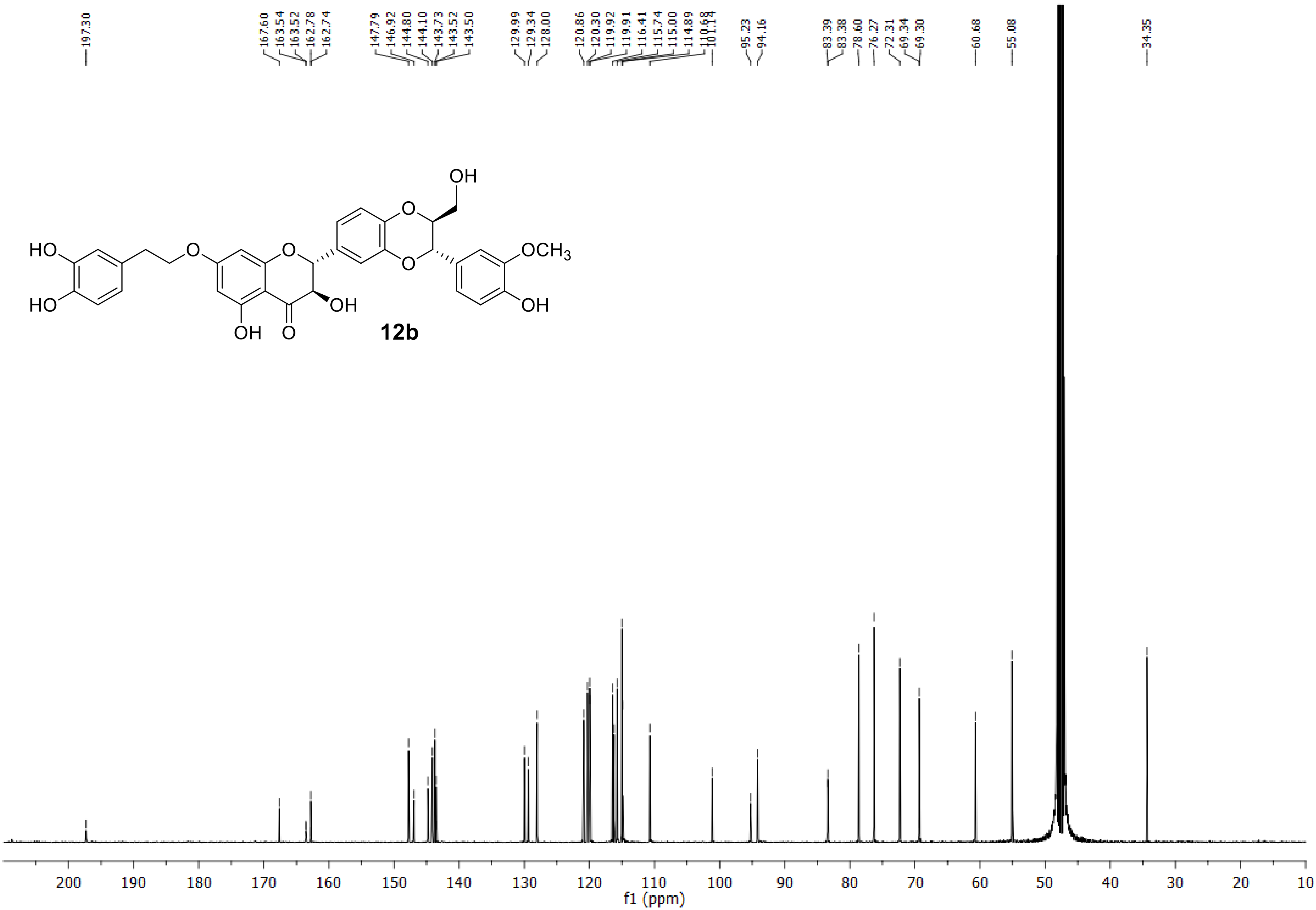

197.30
167.60
163.54
163.52
162.78
162.74
147.79
146.92
144.80
144.10
143.73
143.52
143.50
129.99
129.34
128.00
120.86
120.30
119.92
119.91
116.41
115.74
115.00
114.89
110.68
101.14
95.23
94.16
83.39
83.38
78.60
76.27
72.31
69.34
69.30
60.68
55.08
34.35
HO
HO
O
O
OH
O
O
OCH3
OH
OH
OH
O
12b
200 190 180 170 160 150 140 130 120 110 100 90 80 70 60 50 40 30 20 10
f1 (ppm)

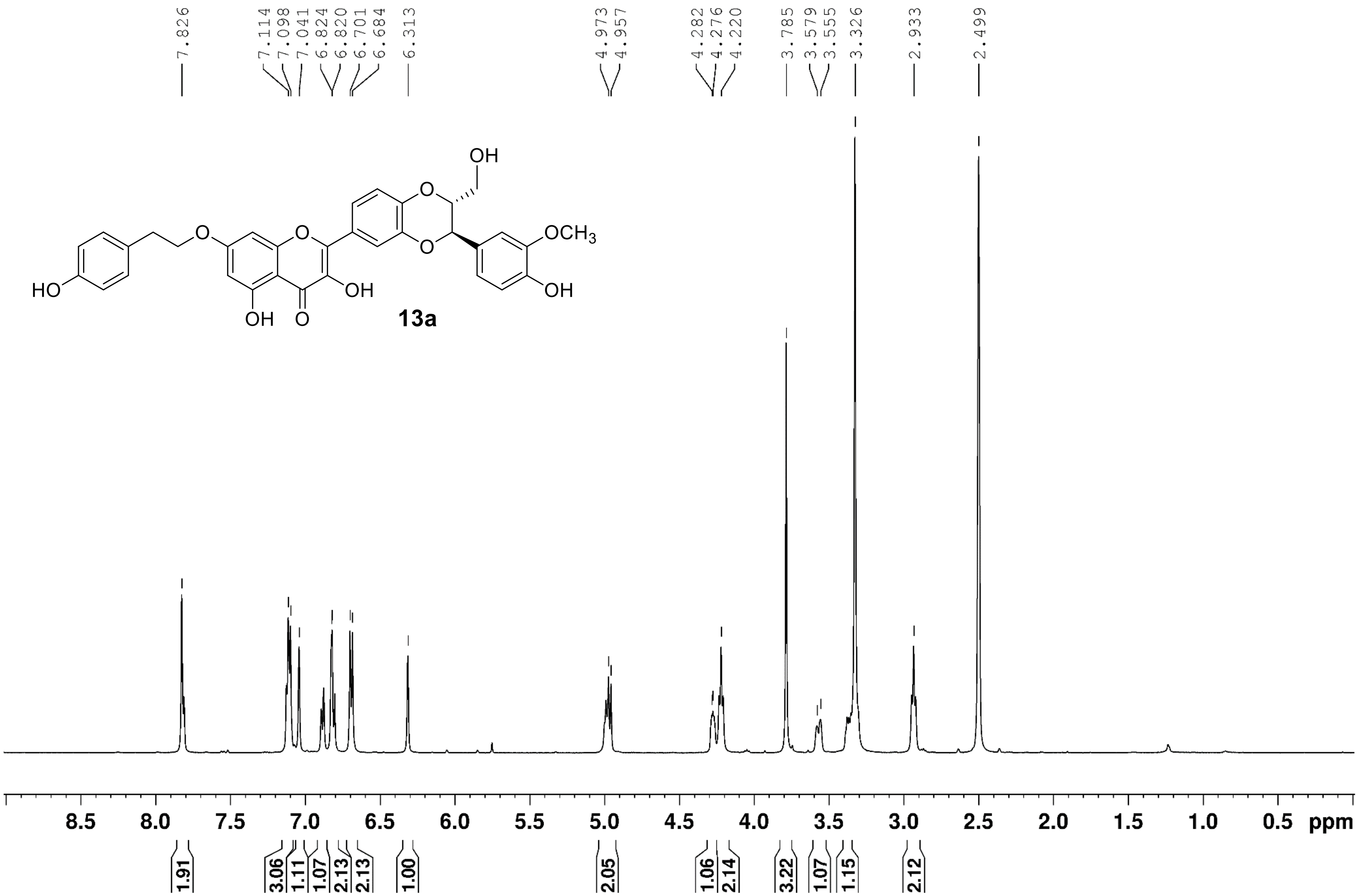
7.826
7.114
7.098
7.041
6.824
6.820
6.701
6.684
6.313
4.973
4.957
4.282
4.276
4.220
3.785
3.579
3.555
3.326
2.933
2.499
OH
O
O
O
O
OCH3
HO
OH
OH
OH
O
13a
8.5
8.0
7.5
7.0
6.5
6.0
5.5
5.0
4.5
4.0
3.5
3.0
2.5
2.0
1.5
1.0
0.5
ppm
1.91
3.06
1.11
1.07
2.13
2.13
1.00
2.05
1.06
2.14
3.22
1.07
1.15
2.12

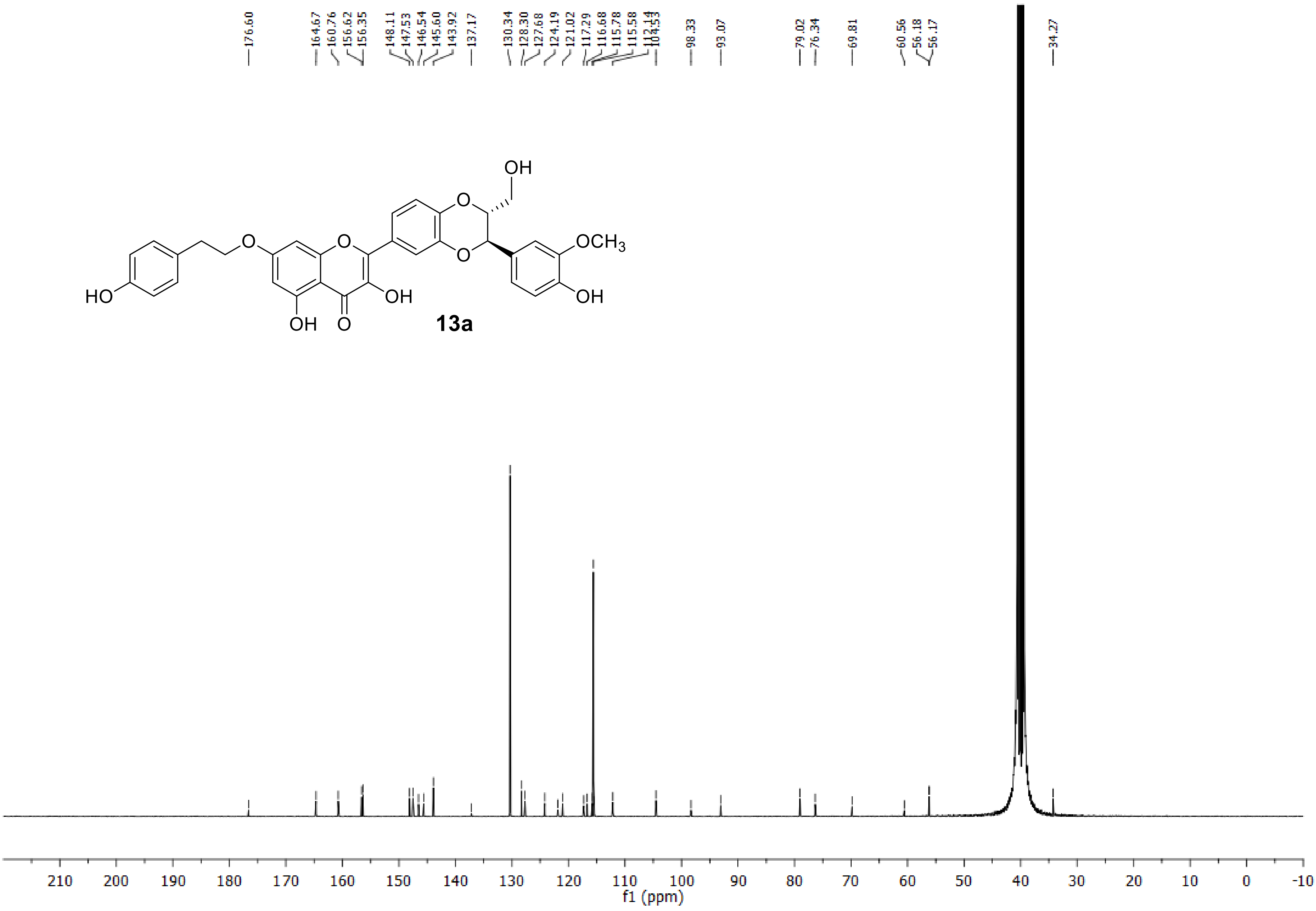
176.60
164.67
160.76
156.62
156.35
148.11
147.53
146.54
145.60
143.92
137.17
130.34
128.30
127.68
124.19
121.02
117.29
116.68
115.78
115.58
112.14
104.53
98.33
93.07
79.02
76.34
69.81
60.56
56.18
56.17
34.27
OH
O
O
O
OCH3
HO
OH
OH
OH
O
13a
210 200 190 180 170 160 150 140 130 120 110 100 90 80 70 60 50 40 30 20 10 0 -10
f1 (ppm)

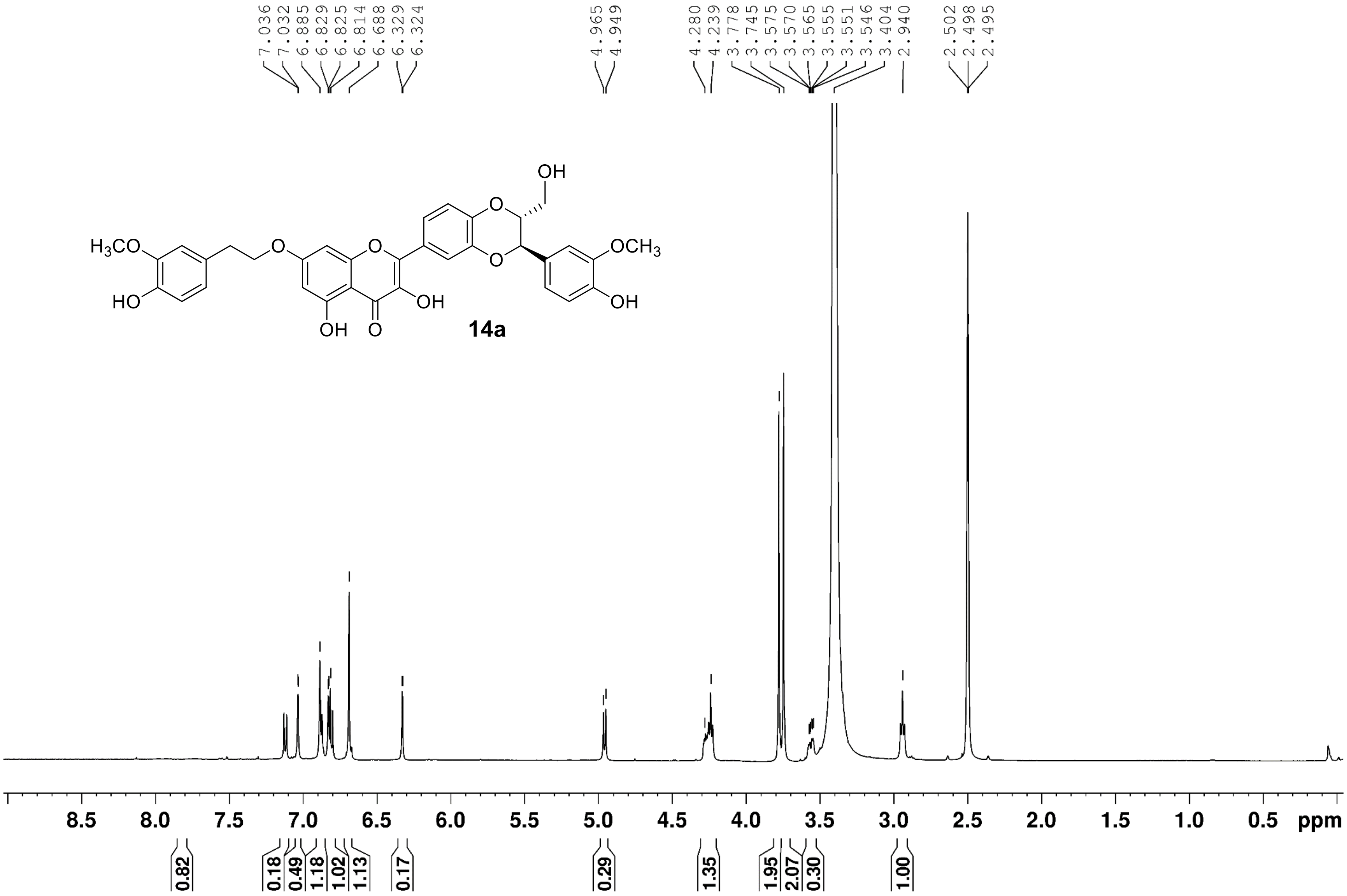
7.036
7.032
6.885
6.829
6.825
6.814
6.688
6.329
6.324
4.965
4.949
4.280
4.239
3.778
3.745
3.575
3.570
3.565
3.555
3.551
3.546
3.404
2.940
2.502
2.498
2.495
OH
H_3CO
O
O
O
O
OCH_3
HO
OH
OH
OH
O
14a
8.5
8.0
7.5
7.0
6.5
6.0
5.5
5.0
4.5
4.0
3.5
3.0
2.5
2.0
1.5
1.0
0.5
ppm
0.82
0.18
0.49
1.18
1.02
1.13
0.17
0.29
1.35
1.95
2.07
0.30
1.00

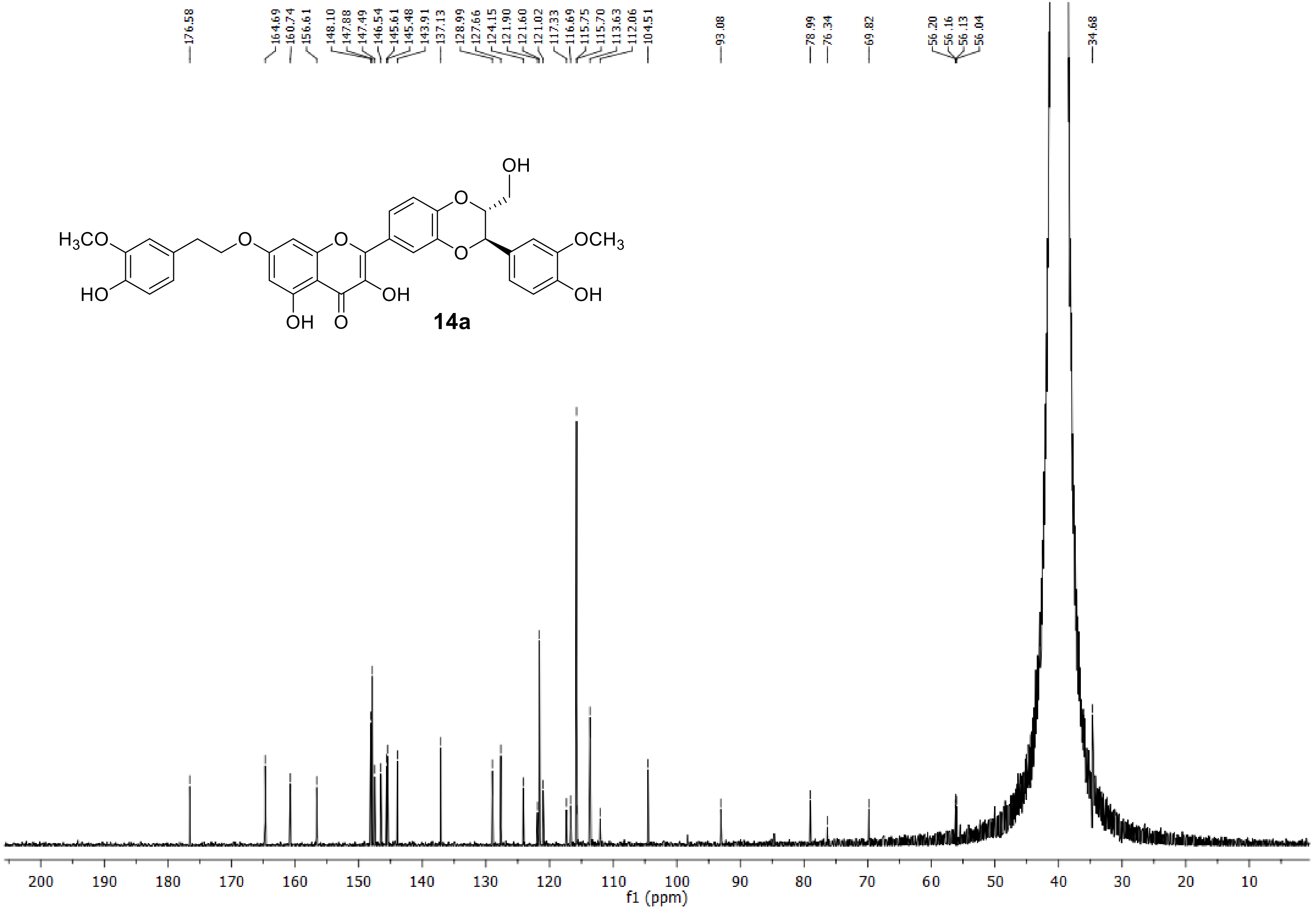
176.58
164.69
160.74
156.61
148.10
147.88
147.49
146.54
145.61
145.48
143.91
137.13
128.99
127.66
124.15
121.90
121.60
121.02
117.33
116.69
115.75
115.70
113.63
112.06
104.51
93.08
78.99
76.34
69.82
56.20
56.16
56.13
56.04
34.68
OH
O
O
H3CO
O
O
OCH3
HO
OH
OH
OH
O
14a
f1 (ppm)

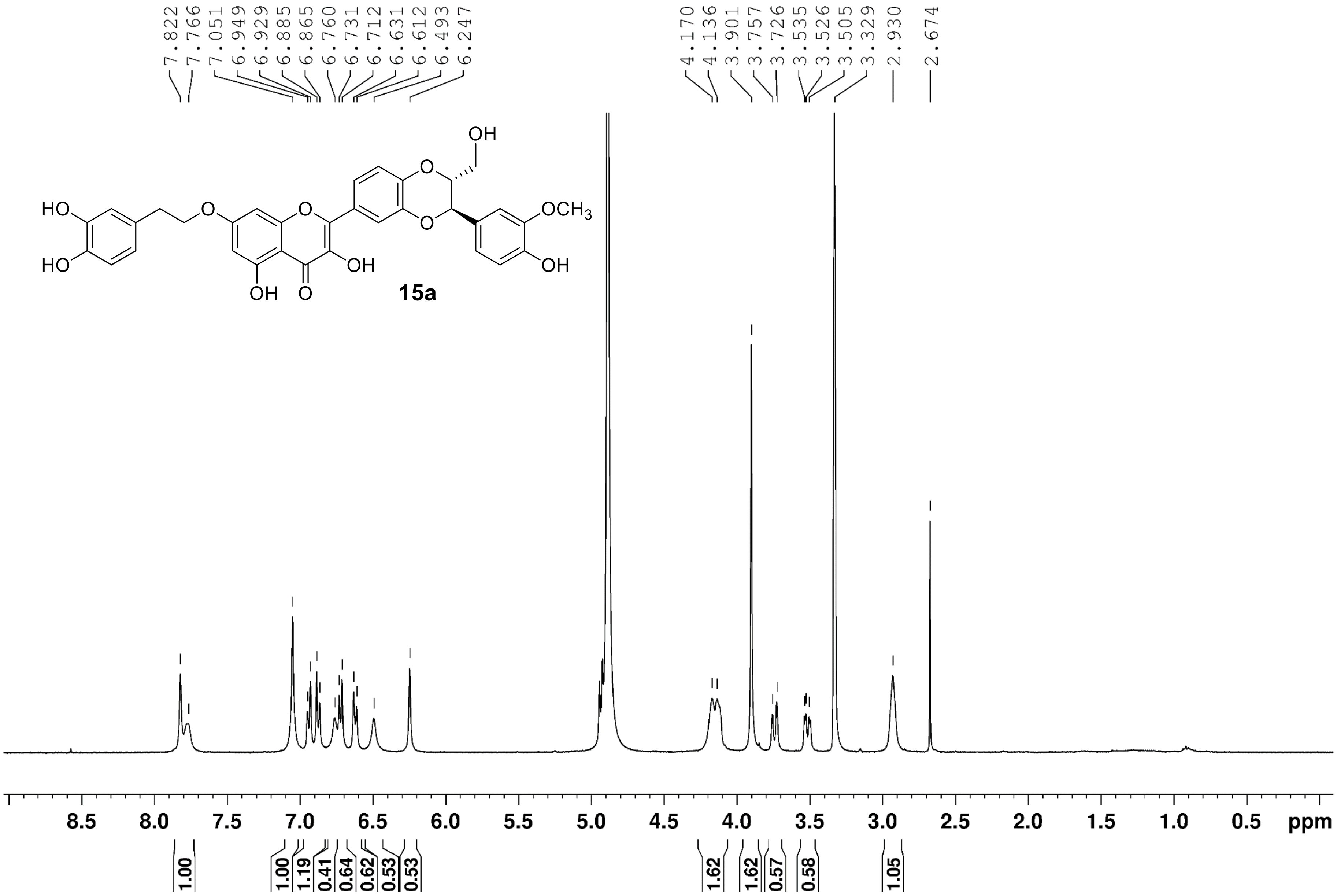
7.822 7.766 7.051 6.949 6.929 6.885 6.865 6.760 6.731 6.712 6.631 6.612 6.493 6.247
4.170 4.136 3.901 3.757 3.726 3.535 3.526 3.505 3.329 2.930 2.674
HO
HO
O
O
OH
OH
O
O
O
OH
OCH3
OH
15a
8.5 8.0 7.5 7.0 6.5 6.0 5.5 5.0 4.5 4.0 3.5 3.0 2.5 2.0 1.5 1.0 0.5 ppm
1.00 1.00 1.19 0.41 0.64 0.62 0.53 0.53 1.62 1.62 0.57 0.58 1.05

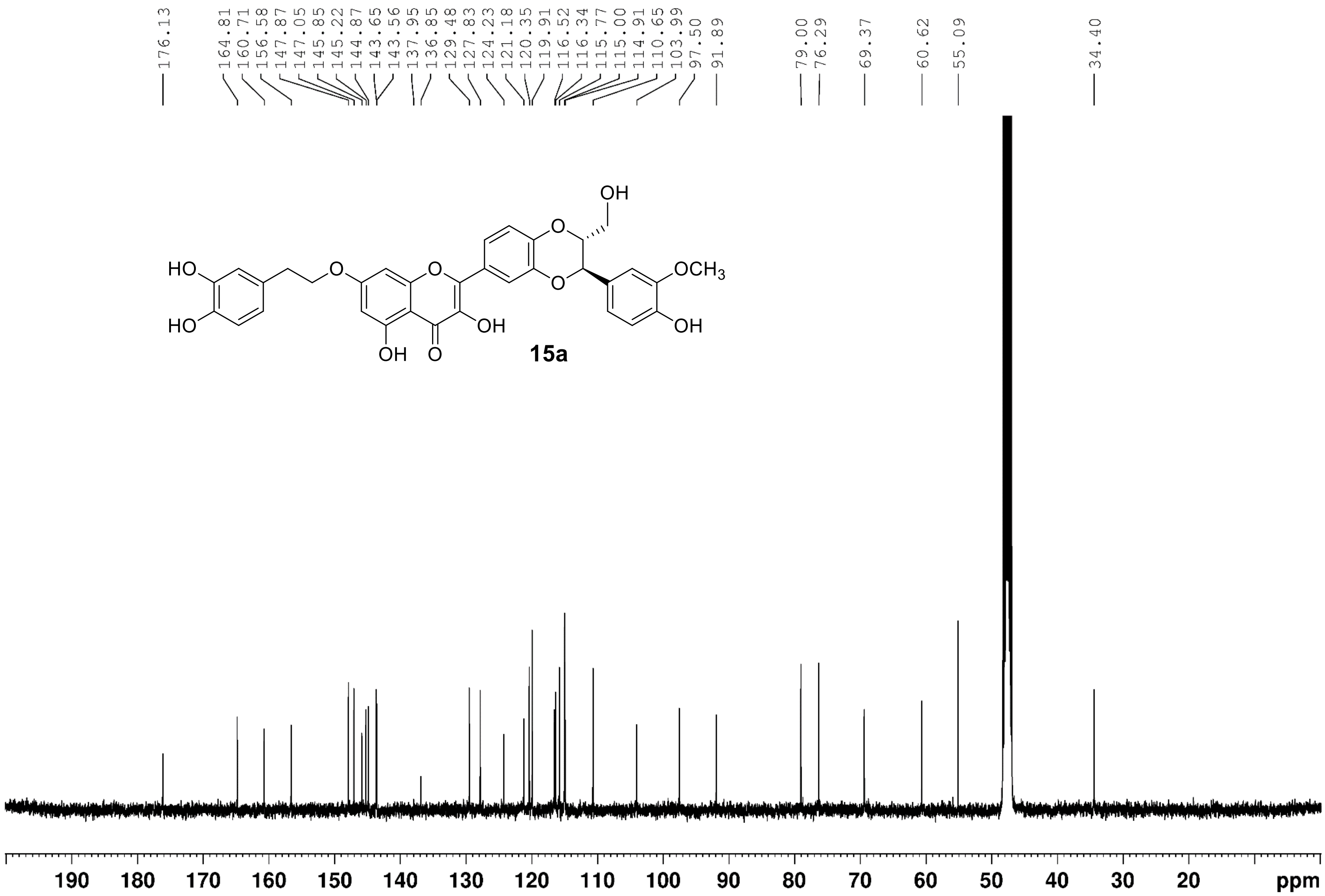
176.13
164.81
160.71
156.58
147.87
147.05
145.85
145.22
144.87
143.65
143.56
137.95
136.85
129.48
127.83
124.23
121.18
120.35
119.91
116.52
116.34
115.77
115.00
114.91
110.65
103.99
97.50
91.89
79.00
76.29
69.37
60.62
55.09
34.40
HO
HO
OH
OCH$_3$
OH
OH
OH
15a
190 180 170 160 150 140 130 120 110 100 90 80 70 60 50 40 30 20 ppm
